# Supplementary material for: Identification of photoperiod-induced specific miRNAs in the adrenal glands of Sunite sheep (Ovis aries)
Source: Front Vet Sci. 2022 Jul 22;9:888207. doi: 10.3389/fvets.2022.888207 (PMC9354845; doi:10.3389/fvets.2022.888207)
Supplement: Supplementary file 1 [file Data_Sheet_1.ZIP › Supplementary file/miRDeep2 file.zip!/miRDeep2 file/Know_Structure/SPLP42A3/oar-mir-539.pdf]

miRBase precursor : oar-mir-539
Total read count : 181
oar-miR-539-5p read count : 1
oar-miR-539-3p read count : 180
remaining reads : 0

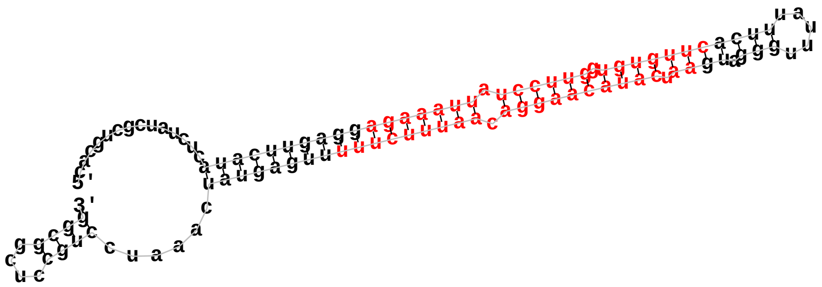

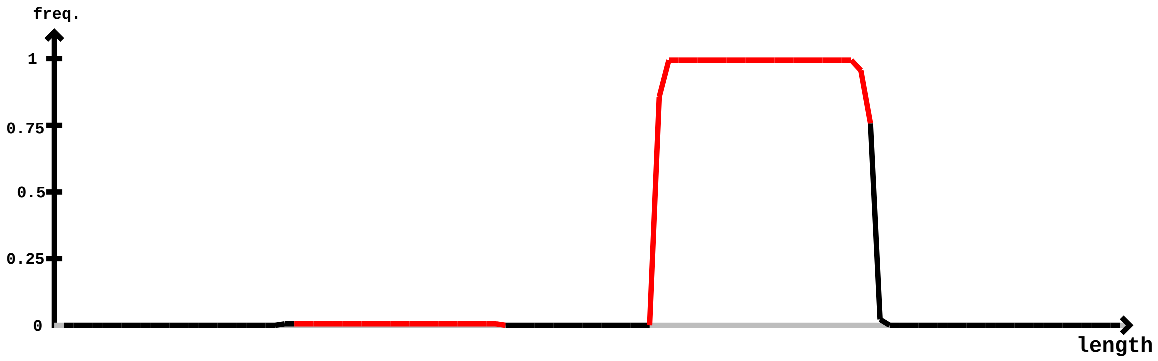


```
                                                                                       oar-miR-539-3p
                                        oar-miR-539-5p
5'- cacgucgcuaucucauacuugaggagaaauuauccuugguguguucacuuuauuugggaugaaucauacaaggacaauuucuuuuugaguaucaaauccugccucggcgg   -3'    exp
    ..............(((((((((((((((((.((((((.(((((((((((.....))).)))).))))))))))).))))))))))))))))......((((....))))    reads      mm         sample
    ......................ggagaaauuauccuugguguguu.................................................................    1          0          seq
    .............................................................aaucauacaaggacaauuucu............................    7          0          seq
    .............................................................aaucauacaaggacaauuucuu...........................    36         0          seq
    .............................................................aaucauacaaggacaauuucuuu..........................    109        0          seq
    .............................................................aaucauacaaggacaauuucuuuu.........................    3          0          seq
    ..............................................................aucauacaaggacaauuucuuu..........................    24         0          seq
    ..............................................................aucauacaaggacaauuucuuuu.........................    1          0          seq
```
